# When do traumatic experiences alter risk-taking behavior? A machine learning analysis of reports from refugees

**DOI:** 10.1371/journal.pone.0177617

**Published:** 2017-05-12

**Authors:** Mareike Augsburger, Thomas Elbert

**Affiliations:** 1 Department of Psychology, University of Konstanz, Konstanz, Germany; 2 NGO vivo international e.V., Allensbach, Germany; 3 Department of Psychology, University of Zurich, Zurich, Switzerland; Stellenbosch University, SOUTH AFRICA

## Abstract

Exposure to traumatic stressors and subsequent trauma-related mental changes may alter a person’s risk-taking behavior. It is unclear whether this relationship depends on the specific types of traumatic experiences. Moreover, the association has never been tested in displaced individuals with substantial levels of traumatic experiences. The present study assessed risk-taking behavior in 56 displaced individuals by means of the balloon analogue risk task (BART). Exposure to traumatic events, symptoms of posttraumatic stress disorder and depression were assessed by means of semi-structured interviews. Using a novel statistical approach (stochastic gradient boosting machines), we analyzed predictors of risk-taking behavior. Exposure to organized violence was associated with less risk-taking, as indicated by fewer adjusted pumps in the BART, as was the reported experience of physical abuse and neglect, emotional abuse, and peer violence in childhood. However, civil traumatic stressors, as well as other events during childhood were associated with lower risk taking. This suggests that the association between global risk-taking behavior and exposure to traumatic stress depends on the particular type of the stressors that have been experienced.

## Introduction

Risk-taking behavior describes behaviors involving the opportunity for both potential loss and reward with an uncertain outcome probability, such as drug consumption, delinquency, unhealthy eating habits or unprotected sex [1]. Numerous studies have identified exposure to traumatic events and subsequent symptoms of posttraumatic stress disorder (PTSD) as risk factors for increased risk-taking behavior [for reviews see 1, 2, 3]. For instance, both a history of different types of childhood maltreatment and lifetime traumatic experiences had a cumulative effect on risk-taking behaviors such as substance abuse and criminality [e.g., 4]. Childhood maltreatment was also a predictor of elevated risky sexual behavior during both adolescence [e.g., 5] and adulthood [e.g., 6], with sexual abuse as a prominent risk factor [e.g., 7, 8]. Moreover, depression symptoms mediated these associations in adolescents who had been abducted or threatened by armed groups in northern Uganda [9]. In their review, Ben-Zur and Zeidner [1] concluded that elevated risk behavior might arise from exposure to life-threatening events. They also discussed different models explaining increased risk-taking behavior after trauma: Some argue that this might be a maladaptive coping strategy in order to overcome negative affect arising after being victimized. Cognition-based models consider modified information processing with respect to the evaluation of risk. Neuroscientific approaches emphasize exaggerated amygdalar activation in response to threat, which might result in an impeded capability to control behavior [for details see 1, 2].

The balloon analogue risk task [BART; 10] aims to reflect shifts in real-life risk-taking behaviors by applying a paradigm with decisions made under uncertainty. In this computer-based game, participants inflate a virtual balloon. For each successful pump, they earn a small amount of money, but pumping also increases the probability of the balloon bursting, resulting in the loss of the money earned for this trial. Alternatively, participants can cash out and proceed to the next trial, thereby keeping the money they have accumulated thus far. Thus, inflation increases the chances of financial gain, but at the cost of an increased risk of losing the money. This reflects the definition of risk behavior with an outcome of potential reward or loss. Higher numbers of adjusted pumps–that is, the number of pumps in trials without a burst–are considered to be more prone to risk-taking [10]. So far, the studies that used BART to assess risk behavior in individuals who have experienced traumatic stress have yielded ambivalent results. Crack- or cocaine-dependent patients with comorbid PTSD presented a greater number of adjusted pumps than patients without PTSD [11]. Furthermore, higher risk behavior in the BART was related to experienced child abuse, and mediated associations to self-reports of increased HIV risk behavior [12]. However, other studies found the opposite pattern of results. For instance, young adults with a history of child abuse took significantly less risk in the BART than participants without child abuse [13]. In a study by Woerner, Kopetz [14], there was no association between pumps in the BART and child abuse in a sample of adult substance abuse patients. Maner, Richey [15] yielded evidence that trait anxiety and worries in healthy undergraduate students that could not be traced back to negative affect were associated with risk-avoidant behavior in the BART.

In light of the heterogeneous results of previous studies, it is possible that specific types of child abuse and traumatic events differentially impact risk behavior. For instance, Moore, Overstreet (8) demonstrated in undergraduate college students that interpersonal events such as physical or sexual traumatic experiences were related to increased risky sexual behavior. In contrast, non-interpersonal events, such as car accidents, were less related to risky behavior.

Since refugee populations present high rates of exposure to traumatic events and subsequent PTSD compared to the general population [16, 17] and are also frequently affected by experiences of childhood maltreatment [18], they may be especially prone to risk-taking behavior. With respect to the current refugee crisis [19], assessing predictors of risk-taking behavior in displaced individuals is essential. However, literature regarding risk behavior among refugee populations is sparse. In a longitudinal study of Cambodian families who resettled in Canada, adolescents whose families were less exposed to pre-migratory political violence and war reported involvement in risk behavior, whereas those who were highly exposed reported less involvement [20]. In contrast, in a cross-sectional study with Cambodian refugee youths, direct and witnessed lifetime exposure to violence was related to higher engagement in risk behavior [21]. Regarding adult individuals, no study has assessed risk behavior in response to trauma exposure in displaced persons.

The objective of the current study was to examine whether specific types of trauma exposure, including those of war and torture with subsequent symptoms of PTSD and depression, were followed by an increase in risk behavior as measured by the BART in displaced individuals from various countries who have resettled in Germany. Taking into account the inconsistent results and the heterogeneous group of individuals regarding traumatic experiences, we chose to apply a novel approach for data analysis–a machine-learning procedure. This has several advantages over more conventional linear modeling techniques: Otherwise undetectable complex non-linear associations between predictor and outcome variables can be modeled in a non-parametric regression-like fashion. Additionally, a large number of predictor variables can be tested simultaneously, even in comparatively small samples. Both would not have been possible using conventional statistical methods. In contrast to these methods, in which an a priori defined theoretical model is tested and evaluated by means of *p*-value based significance, machine learning offers a completely different data-driven approach: By means of successively updating a model, the computer sequentially detects the relation in the data [22]. Since machine-learning algorithms may lead to overfitting, performance is evaluated by predicting the response variable on an independent new data set that has not been used before during the process of model building.

Stochastic gradient boosting machines (GBM) refer to a specific type of machine-learning algorithms applying decision trees [23]. For modeling the response variable, the predictor space is successively partitioned according to a set of if-then statements until homogenous response regions have been identified. Splits of predictor variables take place when minimal prediction error is reached [24, 25]. In this manner, a decision tree grows, which predicts the outcome variable by descending the leaves until the terminal nodes are reached. GBM are a powerful and highly robust modeling tool that can handle missing, non-normal data and can deal with predictor noise [25, 26]. Regarding accuracy, boosting in comparison to other machine learning approaches was superior across different research fields such as epidemiology [26]. Similar machine-learning techniques, such as random forests, have been already successfully applied to psychological topics [e.g., 27, 28].

Since only a small dataset was available for model building, the current study aims to give a first insight into the differential impact of types of traumatic stressors on risk-taking behavior rather than determining specific associations between predictors and the outcome.

## Materials and methods

### Procedure

Participants were recruited at the Centre of Excellence for Psychotraumatology, University of Konstanz. The center provides a trauma-related mental health service for refugees. Participants were recruited in the refugees’ accommodation centers. Moreover, individuals asking for psychological help who had contacted the center were also invited to participate in the study. Questionnaires were applied in semi-structured interviews in German or English and, if necessary, with the help of trained translators (in 75% of cases). Interpreters were trained to translate literally and word-by-word in the first person. Experienced clinical psychologists or M.Sc. Psychology students under supervision conducted the interviews. After the end of the semi-structured interview, a computer-based task to measure risk behavior was applied in a separate laboratory room.

Participants received reimbursement for travel expenses as well as the monetary reward obtained in the computer task. If necessary, a psycho-diagnostic report was provided and further treatment arranged. The Ethics Committee of the University of Konstanz approved the study. All participants provided their written informed consent. See Augsburger, Dohrmann [29] for details regarding further interview modalities and procedures.

### Measures

#### Socio-demographic variables

Age, sex, level of education, country of origin, and family status, among others, were measured, but are not further reported here [see 29].

#### Childhood maltreatment

Experiences of child abuse were assessed using the KERF [30], a German short version of the modified adverse childhood experiences scale with 20 items [MACE, 31]. The KERF has ten subscales and is coded dichotomously (yes/no). In order to reduce the number of predictors but to be able to differentiate between different types of child abuse, categories were created as follows: sexual violence, physical violence (subscales physical violence by parents as well as by siblings), emotional violence (subscales emotional violence by parents as well as by siblings), witnessed violence (subscales witnessed violence towards parents as well as siblings), emotional neglect, physical neglect and peer violence. Items were summed within each category, ranging between 0–10 for sexual violence, emotional neglect, physical neglect and peer violence, and 0–20 for physical violence, emotional violence and witnessed violence.

#### Exposure to war and torture

A shortened version of the ViVo checklist for exposure to war, terror and torture experiences was applied [32]. The checklist asks for different lifetime experiences related to torture (19 items), such as “*Have you suffered sham executions–that is*, *people acted as if they would kill you immediately*?” and war (6 items), such as “*Have you experienced violent house searches or anything similar*?*”* Answers were dichotomously coded (yes/no) and summed to produce a total score ranging between 0–25. The checklist has been successfully applied in refugee populations [33].

#### Lifetime traumatic events

Lifetime traumatic events not covered by the vivo checklist, such as natural disasters, domestic violence or traffic accidents were assessed by means of the shortened event list of the Posttraumatic Diagnostic Scale [34]. Items were coded dichotomously (yes/no) and summed up to receive a score between 0–8.

#### Symptoms of PTSD

PTSD symptom severity according to DSM-IV criteria [35] was assessed by means of the Posttraumatic Stress Symptoms Interview (PSS-I), an interview version of the Posttraumatic Diagnostic Scale [36–38]. The frequency of symptoms was assessed on a four-point Likert scale from 0 (*not at all*) to 3 (*more than 5 times a week*). Symptom scores were summed, with a possible maximum of 51. Cronbach alpha was .96 for the current study.

#### Symptoms of depression

Depression symptoms were assessed using the Patient Health Questionnaire [PHQ-9, 39]. The frequency of symptoms was assessed using nine items based on DSM-IV criteria on a four-point Likert scale from 0 (*not at all*) to 3 (*almost every day*). Scores were summed, with a possible maximum of 27. The PHQ-9 has excellent psychometric properties and can establish the diagnosis of major depressive disorder according to DSM-IV [40]. Cronbach’s alpha was .92 for the current study.

#### Risk-taking behavior

Risk-taking behavior was measured using the BART [10]. Participants received two euro cents for each successful pump. In total, there were 30 balloons. Following previous studies, the bursting point was random between the first and the last pump, with a probability of 1/128 at the time of the first pump, 1/127 for the next pump, and so on.

The BART corresponds to self-reports of real-life risk behaviors in both adolescents [41, 42] and adults [10, 43]. Moreover, it presents convincing test-retest characteristics [44, 45]. The software *The Psychology Experiment Building Language* was used for implementation [46, 47]. Instructions for the BART were read aloud by the translator in the respective language. In addition, the game interface as shown on the screen was explained. Those participants who were not familiar with the use of a computer were instructed on how to handle the computer mouse.

### Participants

The sample was a subsample of another study reported in Augsburger, Dohrmann [29]. In the current study, 56 participants were enrolled. Inclusion criteria were legal age and no signs of acute drug or alcohol intoxication or of psychosis. One individual had to be excluded from data analysis, as he did not understand the instructions of the computer game and pressed the button until each of the balloons had burst. For a second participant, a mean of only the first ten trials was calculated, as he did not want to play further.

The majority of participants were male (89%). Participants’ age was between 19 and 53 years, with a mean of 29.5 (SD = 8.1, median = 28.5). The mean years of education was 9.4 (SD = 4.2), and education largely varied between 0 and 16 years (median = 11). The most frequent country of origin was Afghanistan (*n* = 10), followed by Syria and Gambia (each *n* = 6), and Nigeria and Iran (each *n* = 5). *N* = 4 participants originated from Iraq, *n* = 3 from Sri Lanka. Other countries were Cameroon, Togo, Sudan, Pakistan, and Eritrea (each *n* = 2), and India, Macedonia, Serbia, Somalia, Albania and Bosnia-Herzegovina (each *n* = 1). Most participants were single (n = 33) or in a relationship (n = 18). *N* = 4 were divorced.

### Data analysis

The dataset was randomly split into two sets: A training set in order to build the model (70% of the data) and an independent test set to evaluate prediction accuracy. T-distribution was chosen as loss function, since its heavier tails make it more suitable for small samples [48]. The optimal number of degrees of freedom was systematically varied until the best fit was reached. Using tenfold cross-validation with three repetitions, the model was tuned by testing the performance of varying parameters following the suggestions of Elith, Leathwick (24) for small datasets: Slow learning rates (shrinkage) were chosen (.05, .01, .005, 001) in order to allow enough trees to grow. Tree complexity was set low, beginning with tree stumps and allowing up to three-way interactions between predictors. In order to avoid overfitting, we first fitted with 200 trees and went up to 2000 in steps of 200. The bag fraction–that is, the randomly chosen subsample of the data set used at each set of iteration–was set to .5. Minimum observations per node were varied between 4 and 5. The best-tuned model with lowest root mean squared error (RMSE) achieved by cross-validation was selected. Its prediction accuracy was tested on the remaining set not used for model building. Additionally, R^2^ (squared correlation between observed and fitted values) was calculated.

Exposure to different types of childhood maltreatment, experiences of war and torture as well as lifetime traumatic events and symptoms of depression and PTSD were entered as predictors. Since age and education varied in the study, these variables were also included as predictors. The contribution of each predictor to the overall model was determined by assessing the relative predictor importance following Friedman and Meulman (26): The number of times a variable had been used for splitting was weighted by the squared improvement to the overall model after each split, averaged over trees. This value was scaled, being summed up to 100 for all predictors. In order to visualize the results of GBM, partial dependency plots were applied, presenting the association between a single predictor variable and the outcome by taking into account the average effect of all other predictor variables [24, 49]. The strength of interaction was assessed by calculating Friedman’s H, which quantifies nonlinear interactions between variables on a scale between 0 and 1. The entire procedure was computed several times with different random data splits. Data analysis was carried out within the R environment using the packages *gbm* and *caret* [50, 51].

## Results

All participants had experienced a minimum of one traumatic event, and the overall majority of 93 percent had been exposed to various forms and frequencies of organized violence. The mean exposure to types of torture and war events (vivo checklist) was 7.9 (SD = 6.5, median = 5); the mean exposure in the PSS-I event checklist was 3.3 (SD = 1.4, median = 3).

Childhood maltreatment measured by the KERF was generally high and had been experienced by 94% of participants, but the types presented a very heterogeneous pattern. Physical abuse was most common (85%; mean = 7.9, SD = 5.9, median = 6.6), followed by emotional abuse (65%; mean = 4.8, SD = 4.8, median = 3.3). Peer violence, emotional neglect, and physical neglect were experienced by half of the participants (54%, 52%, and 50%, respectively; mean peer violence = 3.8, SD = 3.9, median = 3.3; mean emotional neglect = 3.1, SD = 3.5, median = 3.3; mean physical neglect = 2.3, SD = 2.7, median = 1.7). The least frequent adverse experiences during childhood were witnessing an event (37%, mean = 2.5, SD = 3.7, median = 0) and sexual abuse (17%, mean = .4, SD = 1.4, median = 0).

Regarding PTSD diagnosis, 55% fulfilled criteria according to DSM-IV (PSS-I mean = 16.4, SD = 13.3, median = 18). The mean score in the PHQ-9 was 10.9 (SD = 7.8, median = 10), indicating a mild to intermediate severity of depression symptoms.

Risk behavior as measured by the BART had a large range between 3.1 and 77.5 adjusted pumps. The mean number of adjusted pumps was 33.0 (SD = 18.9, median = 28.2).

### Bivariate associations between variables

Correlations between predictor variables were all r < .8, and highest for PTSD severity with depression (r = .75, *p* < .001). Accordingly, multicollinearity was of no concern. As expected, there was no meaningful linear association between adjusted pumps of the BART and respective predictor variables: even the highest correlations that were found (with exposure to torture and war events (r = -.29, *p* = .03) and with sexual abuse (r = .27, *p* = .05)) did not account more than 10% of the variance. S1 Fig in the supporting information demonstrates that the correlation between adjusted pumps and sexual abuse arose from one individual having experienced severe abuse.

### Stochastic gradient boosting machine

The trained model with the lowest RMSE of 15.67 (R^2^ = .53) was chosen, with a shrinkage of .005 and an interaction depth of 2. The model was built with 1800 trees, and there were a minimum of five observations per node. S2 Fig in the supporting information illustrates that faster learning rates (.01, .05) resulted in higher RMSE, especially with increasing numbers of trees. A shrinkage of .001 was generally worse than that of .005.

Applying the model to the test set revealed a RMSE of 18.70, being slightly worse than the best-tuned cross-validated model. R^2^ was .20, indicating sufficient predictive performance. Different data splits did only marginally change these indices.

**Fig 1:**
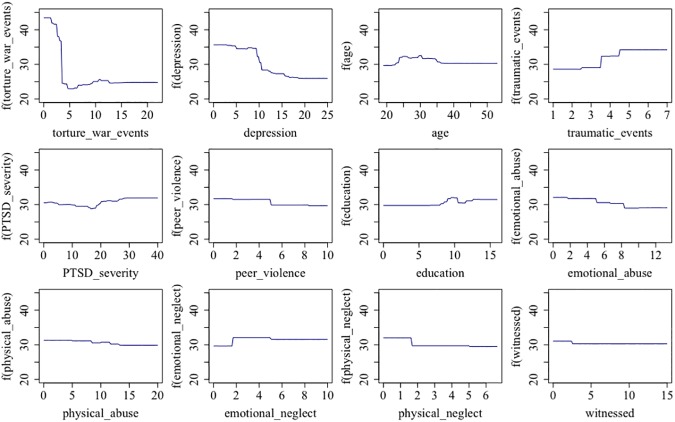
Partial dependency plots for the first 12 variables. For each predictor variable (displayed on the abscissas) the number of adjusted pumps (displayed on the ordinate) is shown as a function of the former.

Exposure to torture and war events was the most important predictor, capturing 30% of the overall importance. The second most important variable in predicting risk behavior was depression symptoms (12%), followed closely by age of participants (11%), and exposure to non-organized traumatic events (9%). PTSD symptom severity (8%), peer violence (6%), level of education (6%) were located mid-range. Exposure to other types of child abuse did not play a major role (all below 5%). Fig 1 visualizes the patterns of association between predictor variables and outcome by means of partial independency plots. A high-risk behaving individual is characterized by no or little exposure to war and torture. However, exposure to other potentially traumatic events seems to increase risk behavior. Regarding mental health, an increasing depression severity led to a substantial drop in risk behavior. Accordingly, PTSD severity had a minimal increase at pronounced levels. Age presented a small peak between 25 and 35 years. High levels of education favored higher risk behavior, though this effect was only visible from around 8 years upwards. Regarding the less substantial impact of child maltreatment, experiences of peer violence, both emotional and physical abuse–and to a lesser extent physical neglect–led to a descent in risk behavior. In contrast, emotional neglect had a positive impact on risk behavior. The relevance of both witnessed events and sexual abuse was negligible.

Two-way interactions were of negligible size being highest for the effect of age x education (Friedman’s H = .08). As effects of two-way interactions were small regarding their overall contribution towards an explanation of variability in the BART, they are not further reported here.

## Discussion

In the current study, high risk-taking behavior was predicted by little or no exposure to organized violence, such as war and torture, but high levels of otherwise experienced lifetime traumatic events, including domestic but also non-interpersonal threats. These results were concordant with Rousseau, Drapeau [20], who reported diminished risk behavior following more frequent exposure to pre-migratory organized violence in adolescent refugees. PTSD symptom severity, which is fueled by both exposure to domestic and to organized violence, contributed little to the variation of pumps in the BART. Increasing symptoms of depression were associated with lower risk behavior, which might be explained by clinical symptoms of fatigue or psychomotor retardation [52].

In contrast to studies that reported increased levels of risk-taking behavior due to general experiences of child abuse as measured by self-reporting, such as increased substance abuse, criminality or risky sexual behavior [4–6], the more fine-grained analysis of different types of childhood maltreatment in our study revealed small but complex impacts on global risk-taking behavior. Apart from emotional neglect, all other types of abuse (peer violence, physical abuse, emotional abuse and physical neglect) were associated with reduced risk behavior.

In accordance with Moore, Overstreet [8], our results point to the importance of taking a differential look at types of traumatic events instead of using a global severity score when assessing a global marker of risk-taking behavior, as with the BART. This point of view can also explain the heterogeneity in results with respect to the few studies that have applied the BART: these studies were either not able to find shifts in risk taking following experiences of child abuse [14], reported increased risk behavior associated with child abuse [12] and PTSD [11], or reported effects in the opposite direction [13].

Beyond this, it might be questioned whether self-reports of specific risk-taking behavior and a global assessment as measured by the BART present two sides of the same coin. Sujan, Humphreys [13] point to the possibility that laboratory-based measurements such as the BART capture momentary decisions, whereas self-reports refer to more general behaviors in real life. As the latter asks for involvement in specific types of risk behavior such as substance dependence or risky sexual behaviors during a defined period of time, it is likely that changes in risk behavior due to traumatic stress are domain specific. Consequently, the BART might assess decision components associated with risky behavior, but not engagement in risk-taking behaviors per se. In light of the evidence that risk aversion in the BART can be traced back to pessimistic risk appraisal in the presence of heightened fear [15], further research is needed to elaborate on the differential impact of types of traumatic stress on risk behavior assessed with the BART.

The current study is the first to assess risk behavior measured by a computer-based task in displaced individuals with extremely high levels of traumatic experiences. Moreover, since there is no research regarding culturally different concepts of risk behavior, education and societal values might vary and have an impact on engagement in risk behavior despite exposure to types of traumatic events.

### Limitations

Originally, GBMs were invented for the purpose of mining high-dimensional data, but were transferred to a small sample size setting in the current study. Consequently, however, only limited data was available to train the model resulting in higher error rates. Prediction accuracy would have been improved if more data had been available. The current model fails to predict very low and high levels of performance in the BART. This also suggests that other parameters that were not taken into account substantially contribute to differentiating between extremely risk-averse and risk-prone individuals. Moreover, the very small sample size of the test set might be biased in terms of generalizability.

Moreover, due to the cross-sectional design of the study, causal directions regarding cause-effect remain to be assessed. Potential influences of participants’ gender and cultural background could not be taken into account due to sample size characteristics. Also, it seems difficult to suggest which real-world risk behavior a lab measurement such as the BART might reflect.

Finally, despite attempts in order to control for overfitting by means of cross-validation procedures and repeated random data splits, this potential weakness of overfitting should be taken into account. The significant drop between cross-validated training data and the test data as well as potentially arbitrary patterns with respect to associations between specific predictors and the outcome (see Fig 1) are highly suggestive of overfitting. Accordingly, strong caution is warranted when interpreting these findings as representative of real-life associations. Rather, the results might be interpreted as indicative of how specific traumatic stressors can differentially modulate subsequent risk-taking behavior, particularly in terms of depression and organized violence as opposed to non-organized violence.

## Conclusions

Altogether, the current study suggests that the experience of organized violence versus domestic violence differentially impacts subsequent performance on a laboratory test for risk-taking behavior such as the BART. Further research with larger sample sizes is needed in order to clarify the specific associations between types of exposure to traumatic events and risk-taking behavior.

## Supporting information

S1 Fig Correlation between adjusted pumps and sexual abuse. (TIF)

S2 Fig Results of the model tuning procedures achieved by repeated cross-validation. Root means squared error (RMSE) for each tuning step is shown on the ordinate for different learning rates (shrinkage), number of trees (boosting iterations), minimum observations per node (n minobsinnode), and interaction depth.
(TIF)

S3 Fig Correlation between predicted and observed values for the test** set. (TIF)
