# Supplementary figures and images for: When do traumatic experiences alter risk-taking behavior? A machine learning analysis of reports from refugees
Source: PLoS One. 2017 May 12;12(5):e0177617. doi: 10.1371/journal.pone.0177617 (PMC5428957; doi:10.1371/journal.pone.0177617)

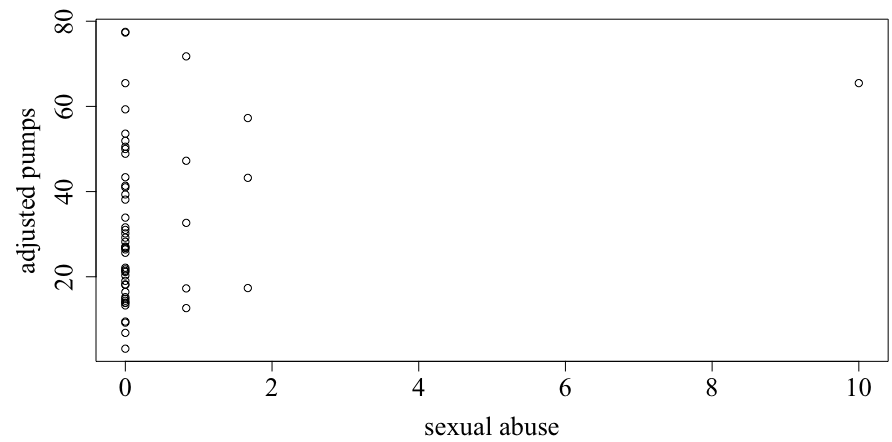

Supplement: S1 Fig — (TIF) [file pone.0177617.s001.tif]

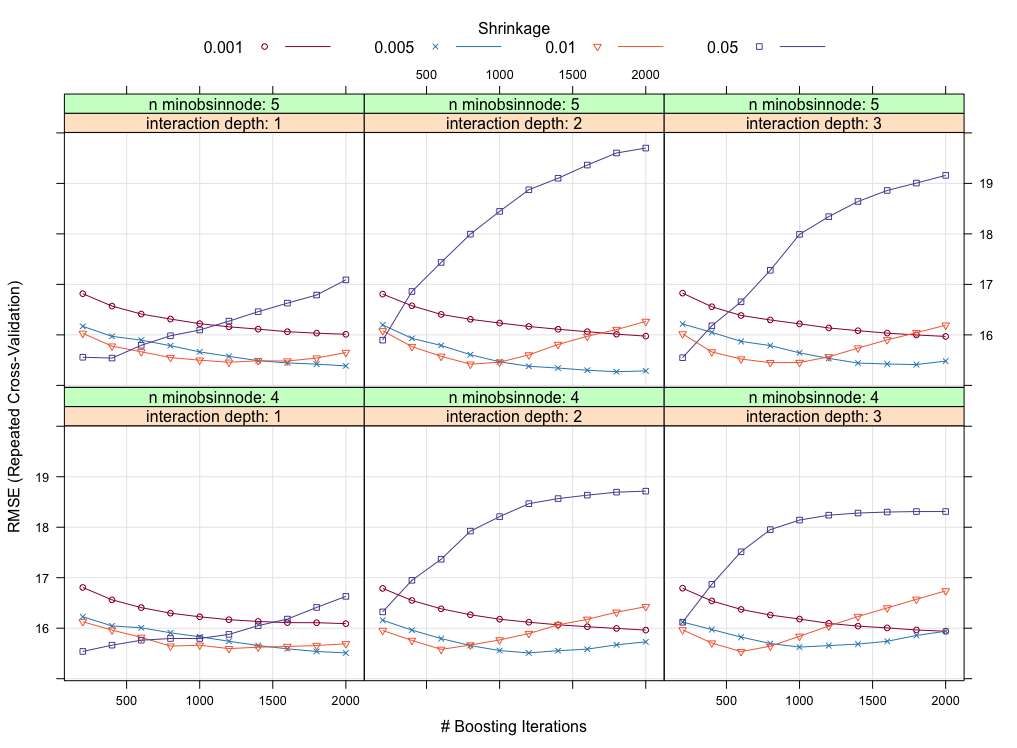

Supplement: S2 Fig — Root means squared error (RMSE) for each tuning step is shown on the ordinate for different learning rates (shrinkage), number of trees (boosting iterations), minimum observations per node (n minobsinnode), and interaction depth. (TIF) [file pone.0177617.s002.tif]

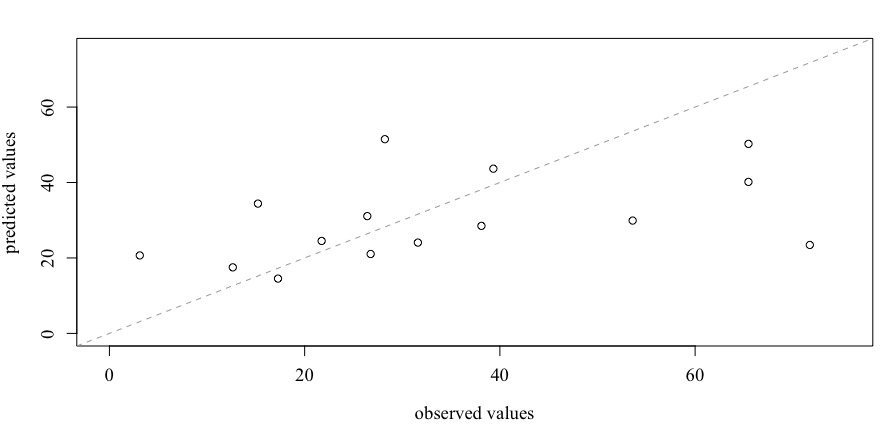

Supplement: S3 Fig — (TIF) [file pone.0177617.s003.tif]
